# Sensitivity to Melody, Rhythm, and Beat in Supporting Speech-in-Noise Perception in Young Adults

**DOI:** 10.1097/AUD.0000000000000621

**Published:** 2019-02-27

**Authors:** Kathryn M. Yates, David R. Moore, Sygal Amitay, Johanna G. Barry

**Affiliations:** 1 Medical Research Council Institute of Hearing Research, School of Medicine, The University of Nottingham, University Park, Nottingham, United Kingdom; 2 Communication Sciences Research Center, Cincinnati Children’s Hospital Medical Center, Cincinnati, Ohio, USA; 3 Nottingham University Hospital Trust, Queen’s Medical Centre, Nottingham, United Kingdom.

**Keywords:** Beat sensitivity, Matrix sentence test, Melody perception, Music experience, Musical training, Rhythm sensitivity, Speech-in-noise perception

## Abstract

**Objectives:**

Musicians appear to have an enhanced ability to perceive speech-in-noise, prompting suggestions that musical training could be used to help people who struggle to communicate in noisy environments. This study assessed the role of sensitivity to beat, rhythm, and melody in supporting speech-in-noise perception.

**Design:**

This is an exploratory study based on correlation. The study included 24 normally hearing young adult participants with a wide range of musical training and experience. Formal and informal musical experience was measured with the training subscale of the Goldsmiths’ Musical Sophistication Index. Speech reception thresholds (SRT) were measured using the Matrix Sentence Test and three different speech-spectrum-shaped noise maskers: unmodulated and sinusoidally amplitude-modulated (modulation frequency, *f*_m_ = 8 Hz; modulation depths: 60 and 80%). Primary predictors were measures of sensitivity to beat, rhythm, and melody. Secondary predictors were pure-tone frequency discrimination and auditory working memory (digit span). Any contributions from these two predictors were to be controlled for as appropriate.

**Results:**

Participants with more musical experience and greater sensitivity to rhythm, beat, and melody had better SRTs. Sensitivity to beat was more strongly linked with SRT than sensitivity to either rhythm or melody. This relationship remained strong even after factoring out contributions from frequency discrimination and auditory working memory.

**Conclusions:**

Sensitivity to beat predicted SRTs in unmodulated and modulated noise. We propose that this sensitivity maximizes benefit from fluctuations in signal-to-noise ratio through temporal orienting of attention to perceptually salient parts of the signal. Beat perception may be a good candidate for targeted training aimed at enhancing speech perception when listening in noise.

## Abstract 2

Supplemental Digital Content is available in the text.

## INTRODUCTION

Everyday communication involves perceiving and understanding speech that is often variably masked by some form of background noise. Depending on the type and level of the masking noise, all listeners may experience difficulty communicating in these conditions, but it is known to be particularly challenging for young children (Klatte et al. 2010), older adults with or without hearing loss (Festen & Plomp 1990; Schneider & Pichora-Fuller 2001; Hall et al. 2002; Füllgrabe et al. 2015), and listeners with some form of language-related developmental disorder (Lagacé et al. 2010; Ziegler et al. 2011). As a consequence, improving capacity to understand speech-in-noise represents one of the single most important goals for auditory habilitation.

Auditory training programs like the Listening and Communication Enhancement (LACE) program (Sweetow & Sabes 2006) have been specifically designed to train speech-in-noise listening. Listeners are typically asked to listen to, and remember, short sentences presented at a fast speaking rate or degraded by background noise. Task difficulty is maintained adaptively. This potentially impacts the listener’s sense of achievement, and task compliance, even in highly motivated adults, can be rather low (Sweetow & Sabes 2010), perhaps contributing to the limited success of the programs (Saunders et al. 2016). It has, therefore, been suggested that musical training could provide a valuable adjunct to these more standard approaches to auditory habilitation, since numerous studies have demonstrated a relationship between musical training and better speech-in-noise perception (e.g., Parbery-Clark et al. 2009b, 2011a; Swaminathan et al. 2015). Furthermore, musical training is inherently engaging and offers many additional social benefits. Patients are, therefore, more likely to comply with long periods of training (Patel 2011; Slater & Kraus 2016).

If musical training is to be used effectively as an auditory habilitation tool, many questions still have to be answered (Coffey et al. 2017). Of particular interest for this study were:

1. Do variations in musical training associate with individual differences in speech-in-noise perception. In other words, is a high degree of musical expertise necessary to observe enhanced speech-in-noise perception?
2. Which perceptual sensitivities, associated with musicality and potentially enhanced through training, are responsible for these improvements?

### Skills Developed Through Musical Training

Music and speech are complex auditory signals that share many properties (Patel 2011). Specifically, both are created by combining basic elements according to a set of rules (Patel 2003) to convey important information via temporal rhythms and pitch pattern changes over time (Kraus & Chandrasekaran 2010).

The basic elements in music are notes, essentially pitch variations, which are combined to form distinctive sequences called “melodies” (Peretz & Zatorre 2005). These may occur alone or in combinations with other melodies. Musicians are known to have enhanced sensitivity to changes in pitch contours in both speech and music (Schön et al. 2004), and their better speech-in-noise perception may reflect their ability to exploit this sensitivity to help segregate the target signal from any background noise (Parbery-Clarket al. 2009a; Zendel & Alain 2009).

Beat and rhythm describe the temporal organization of the notes within a piece of music (Peretz & Zatorre 2005; Schön & Tillmann 2015). Beat is pre-eminent and refers to the isochronous pulse upon which notes of different durations and stresses are superimposed to create rhythm. While speech is not strictly isochronous, much as with music, listeners can perceive and entrain to regularity in speech (Lidji et al. 2011; Schmidt-Kassow & Kotz 2009a). This capacity must play a role in speech perception since disruptions in speech rhythm have a negative impact on intelligibility (Ghitza & Greenberg 2009; Schön & Tillmann 2015).

Reflecting the similarities in the organization of speech and music, as well as findings suggesting links in processing between the two, sensitivity to melody, rhythm and beat are candidate skills for a musical training program aimed at improving speech-in-noise perception. The aim for this study was to objectively assess the role of musical training and experience-related enhancements in sensitivity to melody, beat, and rhythm in supporting speech-in-noise perception.

One of the challenges for addressing our study aim effectively was that musical training is also known to associate with working memory (Chan et al. 1998; Jakobson et al. 2008) and frequency discrimination (Micheyl et al. 2006; Barry et al. 2013). Both these abilities are also thought to be important in supporting speech-in-noise perception (Akeroyd 2008; Parbery-Clark et al. 2009b). Indeed, Kraus et al. (2012) have argued that musicians’ better speech-in-noise perception may derive from their training-related enhancements in auditory working memory. It was, therefore, necessary to include measures of frequency discrimination and auditory working memory to control for any contributions from them to speech-in-noise perception.

There have been many studies investigating the role of musical training in enhancing speech-in-noise listening. Most, if not all, involve participant groups that are defined according to presence or absence of musical training, with expertise being established based on hours of practice per day or years of learning. Sometimes, instrument-specific training is also considered (Slater et al. 2017). Recruitment criteria such as these arbitrarily categorize continuous, multidimensional distributions, taking no account of intermediate levels of experience, different types of training, or underlying differences in untrained musicality. As a result, these studies offer little insight into how much training is required to achieve a change in speech-in-noise perception. To address this limitation, we treated “training” as a continuous variable and applied a correlational design to address our study questions.

### Testing Speech-in-Noise Perception

While the evidence, on balance, suggests musical training enhances speech-in-noise perception (Coffey et al. 2017), some studies have failed to find an effect (Ruggles et al. 2014; Boebinger et al. 2015) or have found an effect that is limited to specific listening conditions (Swaminathan et al. 2015) or even specific tests (Parbery-Clark et al. 2009a). Choice of speech test and masker are, therefore, important considerations.

Speech-in-noise listening in musicians has typically been assessed using open-set sentence test lists from tests like the Quick speech-in-noise test (QuickSIN: Killion et al. 2004) or the Hearing In Noise Test (HINT: Nilsson et al. 1994). These tests may be suitable for studying cross-sectional differences in speech-in-noise listening, but the corpus of sentences available for use is necessarily limited. This means the sentences can be learned over multiple administrations, reducing the test–retest reliability of the test lists and limiting their capacity to reliably measure changes in perception following musical training. Furthermore, sentences can vary in syntactic and semantic complexity both within test lists and across different speech-in-noise tests. As a consequence, in addition to perception, performance will variably reflect influences from individual differences in linguistic or cognitive processing (Parbery-Clark et al. 2009b).

For this study, we wanted a test that focused on sensory, not cognitive, aspects of perception. Additionally, we wanted a test that could be administered multiple times with a high test–retest reliability. It needed to be time efficient, sensitive to potentially quite small differences between individuals, and able to be used with a range of different maskers. The UK Matrix Sentence Test (HoerTech GmbH, Hagerman 1982; Kollmeier et al. 2015) met many of these needs. It is based on 5-word target sentences of the form “Subject[name]–Verb–Object[numeral–adjective–noun],” for example “Nina kept three small desks,” where each word in the sentence is randomly picked from 10 possible options per word position. A speech reception threshold (SRT) is determined adaptively based on performance on each word in the sentence. Each sentence, thus, offers five possible scoring opportunities. The sentences are syntactically and semantically correct, but there are no contextual cues to support word identification. Finally, though the Matrix Sentence Test is supplied with an unmodulated speech-spectrum-shaped noise, it can be used with different maskers.

Amplitude modulation of a speech-spectrum-shaped noise is known to increase the sensitivity of a speech test to individual differences in perception (Wagener & Brand 2005). This extra sensitivity arises because the modulations in the noise introduce brief increases (“dips”) in signal-to-noise ratio (SNR). Listeners vary in their ability to take advantage of these dips, in other words “to dip-listen,” resulting in a greater range of individual SRTs compared with unmodulated noise maskers (Wagener & Brand 2005; George et al. 2007). However, this increased sensitivity comes at a cost in terms of reduced reliability (Wagener & Brand 2005), which is problematic for assessment of treatment-related benefit. We, therefore, needed to identify a modulation depth that would maximize intersubject variability, while minimizing intrasubject variability. To do this, we compared SRTs for two different modulation depths (60 and 80%; Gnansia et al. 2008). We also compared performance on these modulation depths with performance on the unmodulated (“steady state”^1^) masker supplied with the Matrix Sentence Test. This allowed us to derive a measure of modulation masking release separate from any procedural (top-down) influences on task performance (Moore 2012; Dillon et al. 2014).

### Study Aims

In summary, the aim of this study was to assess the role of sensitivity to melody, beat, and rhythm in supporting better speech-in-noise perception. This was done by separately measuring these sensitivities in people with a range of musical experience and using a correlational design to gauge the extent to which musical expertise contributes to speech-in-noise perception. As part of the study, we first determined a depth of amplitude modulation to optimize the sensitivity and reliability of the Matrix Sentence Test for measuring individual differences in speech-in-noise perception.

## MATERIALS AND METHODS

Permission for the study was provided by the Nottingham University Hospital Research Ethics Committee.

### Participants

Native English speakers with a broad range of music abilities (n = 24, 10 male; mean age: 25.9 ± 6.1 years; range: 19 to 40 years) were recruited by means of posters displayed around the University of Nottingham, in the local community, and within the Music Department. All participants had normal hearing (bilateral pure-tone thresholds ≤20 dB HL; octave frequencies 250 Hzto 8 kHz) and a T-score ≥ 80 on the matrix reasoning subtest of the Wechsler Abbreviated Scale of Intelligence (WASI: Wechsler & Chen 1999). Level of music experience was assessed using the Goldsmiths’ Musical Sophistication Index.

### Tests

#### Goldsmiths’ Musical Sophistication Index—Musical Experience

The Musical Training subscale of the Goldsmiths’ Musical Sophistication Index version 0.9 (Gold-MSI: Müllensiefen et al. 2014) consisted of nine statements encompassing both formal instrument training and informal musical experience, such as singing in a choir. Participants provided responses to each statement on a 7-point scale, and these were summed to result in a score ranging from 9 (relatively little training or experience) to 63 (highly trained, with a lot of experience).

Given that the scale captures both formal and informal aspects of musical experience, we refer to “musical experience” rather than “training” when reporting results from it.

#### Digit Span—Auditory Working Memory

The digit span forward and backward subtests from the Wechsler Adult Intelligence Scale (Wechsler 2008) were used to measure auditory working memory. For the forward subtest, strings of 2 to 9 digits were read out loud, with 2 trials for each string length. Participants had to repeat all the digits in the correct order to score a point for that trial. The test terminated when a participant failed on both trials at a given sequence length. The procedure was repeated for the backward test, except that participants had to repeat the digits in reverse order, and there were a maximum eight digits to be recalled.

Scores from the forwards (serial memory) and backwards (executive function; Rosenthal et al. 2006) subtests were summed to obtain a score for auditory working memory. The maximum score from this test was 32.

#### Pure-Tone Frequency Discrimination—Estimation of Sensitivity to Temporal Fine Structure

Frequency discrimination thresholds were obtained using a three-interval, three-alternative, forced-choice procedure (Amitay et al. 2006). Each trial consisted of three tones of 100 ms duration (including 15 ms cosine on/off ramps) with an interstimulus interval of 300 ms. Two tones were identical (“standard” frequency, *f* = 1 kHz), while the third “target” tone had a frequency of *f* + (Δ*f* *f) where Δ*f* was a percentage of *f*. Participants were asked to indicate, by button press, the tone that differed from the other two. An adaptive staircase procedure was used to target the 79.4% correct point of the logistic psychometric function. The starting value for Δ*f* was 0.5 (50%). It was progressively halved until the first reversal. After this, a 3-down-1-up staircase was implemented with a factor of √2.

After 5 practice trials, participants completed 2 tracks of 50 trials each to obtain 2 thresholds, which were averaged.

#### UK-Matrix Sentence Test—Estimation of Speech Reception Threshold in Noise

The UK Matrix Sentence Test (HoerTech GmbH: Kollmeier et al. 2015) was used to determine SRT (SNR equating to 50% correct identification). In this test, five-word sentences of the form Subject[name]–Verb–Object[numeral–adjective–noun] were formed from a closed matrix (Supplemental Appendix, Supplemental Digital Content 1, http://links.lww.com/EANDH/A461) with 10 possible choices for each word category type (e.g., “Nina kept 3 small desks”).

The masker noise supplied with the test was a quasi-stationary speech-spectrum-shaped noise without strong fluctuations where the long-term spectrum of the noise was designed to match the long-term spectrum of the speech material (Kollmeier et al. 2015). The noise was either unmodulated (as supplied) or sinusoidally amplitude-modulated (*f*_m_ = 8 Hz) with modulation depths of either 60 or 80%, chosen from pilot work to span a range of performance. The level for all noise maskers was set at 65 dB SPL (root mean square), and each masker started from a random point within a continuously generated stream. Sentences were presented at an initial level of 75 dB SPL (SNR = +10 dB), which was varied adaptively (Brand & Kollmeier 2002) to target the SRT. After each sentence, the matrix of possible words (Supplemental Appendix, Supplemental Digital Content 1, http://links.lww.com/EANDH/A461) appeared on screen, and participants used a touchscreen or mouse to select the words they had heard. There was no time limit for responses, and no feedback was given.

Participants completed 4 test lists each comprising 20 sentences with each masker (i.e., 4 Lists × 20 Sentences × 3 Masker Conditions = 240 sentences). The masker conditions were delivered in counterbalanced order. Performance tended to be most variable with the first sentence list for each masker condition and to stabilize thereafter. This first list was, therefore, treated as a practice list and, observations from it were excluded from the calculation of the mean SRT for each condition.

#### Musical Ear Test—Melody and Rhythm Sensitivity

Sensitivity to melody and rhythm was assessed using the two subtests of the Musical Ear Test (MET: Wallentin et al. 2010). Both subtests required listeners to compare two phrases and decide whether they were the same or different. The melodic phrases were made up of 3 to 8 tones of sampled piano sounds, while the rhythmic phrases consisted of 4 to 11 sounds created using a wood block percussion instrument. Each subtest comprised 52 trials: 26 “same” trials and 26 “different” trials. The “different” trials contained one deviation, in pitch or rhythm. These deviations varied in ease of detectability, ensuring a test that was sensitive to a wide range of musical abilities.

At the beginning of each subtest, the test was explained. Participants then listened to two example phrase pairs (one same and one different), before completing the subtest. Responses were recorded on an answer sheet for later scoring, and no feedback was given during the test. The maximum score for each subtest was 52.

#### Beat Alignment Test—Beat Sensitivity

Beat sensitivity was assessed using the auditory-only subsection of the Beat Alignment Test (BAT: Iversen & Patel 2008). The test uses 12 musical excerpts (mean length: 15.9 ± 3.1 sec) from three different genres (jazz, rock, and pop orchestral). Five seconds after the onset of each excerpt, a train of beeps (1 kHz pure tones, 100 ms duration) was superimposed that was either on- or off-beat. Participants had to compare the musical excerpt and accompanying train of beeps to decide if the latter was on, or off, the beat of the excerpt. The test comprised 36 trials, 12 on-beat and 6 each of 4 off-beat conditions. The off-beat conditions differed either in tempo (10% too fast or too slow) or in phase (30% ahead of, or behind, the beat). There were three trials per excerpt, one on-beat, one tempo-adjusted, and one phase-adjusted trial. Before testing, participants completed four practice trials. Two were on-beat (the same excerpt with beeps at two different tempi), and two were off-beat (one tempo-adjusted and one phase-adjusted). Participants were instructed to listen only and not to move or tap along to the music. Responses were recorded on an answer sheet, from which the percent correct scores were determined.

### General Procedure

All testing was done in a sound-attenuating booth and lasted approximately 2 hours, with participants taking breaks when needed. Auditory stimuli were presented diotically through Sennheiser HD-25 headphones.

Testing was structured to maintain attention. Participants first completed the Gold-MSI questionnaire and the WASI matrices test. Then, they completed each of the remaining behavioral tasks in the order presented above, with the exception that the Matrix Sentence Tests for the three masker conditions were interleaved between the three musical skills tests. The order of the three masker conditions for the Matrix Sentence Tests was counterbalanced across participants.

#### Analyses

Normality was checked for all predictor and dependent variables using a combination of histograms, probability plots, and Kolmogorov–Smirnov tests. The only variable not normally distributed was frequency discrimination. This reflects the fact that the thresholds were logarithmically scaled. These data were normalized by applying a reciprocal transformation (i.e., new score = 1/original score).

One participant had SRTs that were outside the normal range for speech perception in modulated noise. Preliminary regression analyses with SRT as the dependent variable and each of auditory working memory, frequency discrimination, and musical experience as predictor variables were performed. In all cases, the studentized residuals were greater than 2 for the outlier. This participant was, therefore, identified as a bivariate outlier and removed from all further correlation analyses.

Pearson correlations were performed for the remaining participants to investigate the relationships among the predictor variables (musical experience, frequency discrimination, auditory working memory, and the three musical skills tests). *A priori*, we predicted that more musical experience would be reflected in better performance on all predictor and dependent variables, so all tests were one-tailed.

Given the exploratory nature of the study, the correlations were not corrected for multiple comparisons, although those that would remain significant even with stringent (Bonferroni) correction were identified. Partial correlation coefficients were calculated to examine the relationships between musical skills and SRTs after controlling for frequency discrimination and auditory working memory.

## RESULTS

### Comparison of the Sensitivity and Reliability of the Three Noise Maskers

Table 1 summarizes the SRTs for the three maskers, mean inter- and intra-subject standard deviation and two measures of test reliability: (a) the ratio of inter- and intrasubject variability (test–retest reliability; Wagener & Brand 2005) and (b) intraclass correlation (a measure of the consistency of measurement of SRT for each condition). As expected, lower (better) SRTs were observed with increasing modulation depth. The 80% modulation depth masker resulted in the largest intersubject standard deviation, suggesting this masker was more sensitive to individual differences in speech-in-noise perception than the other two. The 0% modulation depth masker had the lowest mean intraindividual standard deviation suggesting higher reliability of observations between tests. Importantly, the ratios of inter- to intrasubject standard deviation for both the 0 and 80% modulation depth maskers were greater than 2, suggesting both maskers offered reliable measures of SRT (Wagener & Brand 2005). The 80% modulation depth masker was, therefore, better than the 60% modulation depth masker for addressing the key research aims. The data from the latter condition were, therefore, excluded from all further analyses.

**TABLE 1.**
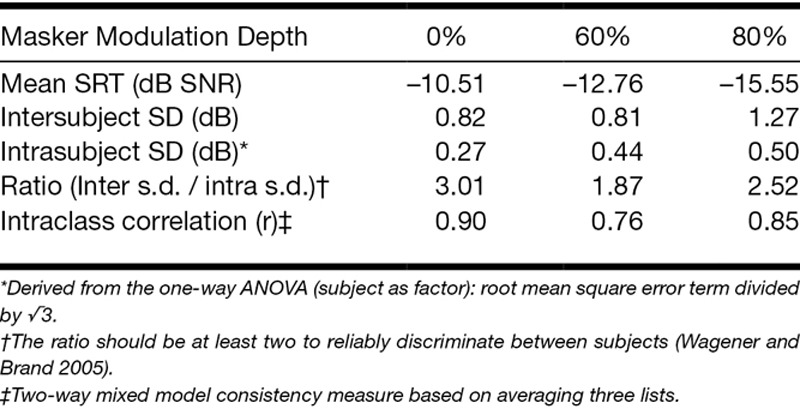
Sensitivity and reliability statistics for the Matrix Sentence Test with the three different noise maskers

### Relationships Among Predictor Variables

Table 2 summarizes group data for the primary and secondary predictor variables. There was a significant skew and kurtosis to the distribution of the frequency discrimination thresholds that, as described above, was corrected using a reciprocal transformation. All other predictor variables showed a normal distribution of responses with little evidence of floor or ceiling effects.

**TABLE 2.**
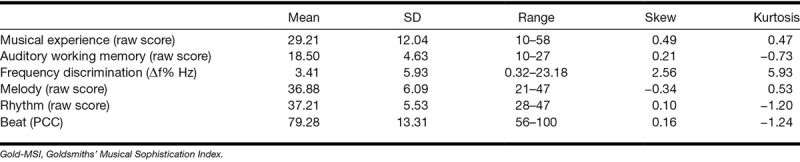
Mean, SD, and range for musical experience (musical training subscale of the Gold-MSI), auditory working memory (digit span forwards + backwards), frequency discrimination, melody, rhythm (Musical Ear Test), and beat (Beat Alignment Test)

**Fig. 1.**
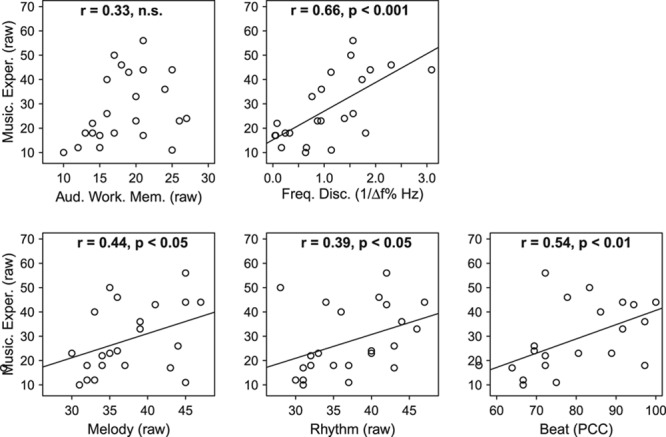
Scatter plots describing the relationships between music experience, the musical related perceptual skills (melody, rhythm, and beat), auditory working memory, and frequency discrimination. Linear regression lines indicate significant association between variables (Table 3).

Table 3 shows the correlations between the variables. Musical experience was positively and significantly associated with frequency discrimination and sensitivity to melody, rhythm, and beat (Figure 1, Table 3). Scores for all music-related measures were widely, and quite evenly, spread across the sample (Figure 1). There was no evidence for a “step” or other nonlinear relationship that might have been predicted by some sort of threshold level of experience or training. Auditory working memory correlated significantly with frequency discrimination and all measures of musical ability, but not with musical experience. Frequency discrimination related to melody and beat, but not rhythm sensitivity. The melody and rhythm subtests of the MET were strongly related to each other and also to auditory working memory. Skill-specific variance is evident in the relationships with other variables: melody was related to the other spectral task (frequency discrimination), while rhythm perception was related to performance on the other slow temporal task (the BAT: beat perception).

**TABLE 3.**
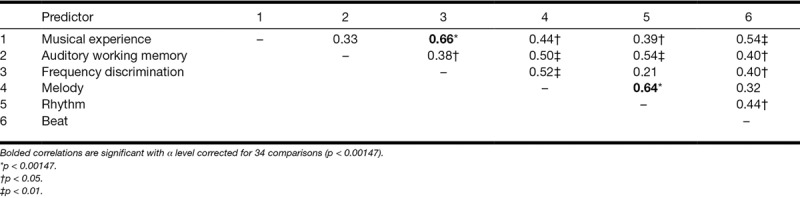
Pearson correlation coefficients (one-tailed) between predictor variables

### Predictors of Speech Reception Thresholds

**Fig. 2.**
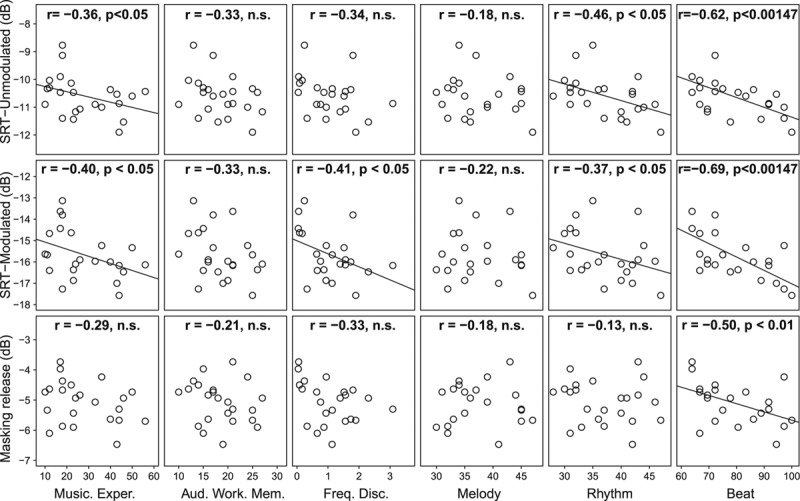
Scatter matrix showing relationships between predictor variables and SRT in unmodulated noise, 80% modulated noise, and masking release [speech reception threshold (SRT) (modulated) − SRT(unmodulated)]. Pearson correlation coefficients (one-tailed) are reported. Correlations that are significant with α level corrected for 34 comparisons are indicated as *p* < 0.00147. Linear regression lines indicate significant association between variables.

Better (higher) scores for some predictor variables were associated with better (lower) SRTs and greater (more negative) masking release (Figure 2).

Musical experience was moderately correlated with SRTs in unmodulated and modulated noise, although the correlations would not have been significant if corrected for multiple comparisons. Nonetheless, the evidence of correlation supports the notion that a link exists between musical experience (formal and informal) and speech-in-noise perception, rather than being restricted to lifelong musicians.

Auditory working memory did not correlate significantly with SRT for either masker condition. Frequency discrimination correlated with SRT in the amplitude-modulated masker condition only.

With respect to the musical skills, melody perception did not significantly correlate with SRTs in either modulated or unmodulated noise. Rhythm perception correlated with SRTs in both conditions, as did beat perception. In this latter case, the correlations were strong and remained significant after correction for multiple comparisons (Bonferroni). Beat perception, unlike rhythm perception, also correlated significantly with modulation masking release.

### Partial Correlations

A level of working memory is required to support performance on both the rhythm and beat tests. Additionally, frequency discrimination abilities are likely to contribute to performance on the beat test since beeps are superimposed on musical melodies. Frequency discrimination and auditory working memory are also known to relate to musical training/experience (Parbery-Clark et al. 2009b) and may contribute to speech-in-noise perception. Partial correlations (Table 4) were, therefore, performed to separate out influences of auditory working memory and frequency discrimination. The association between rhythm and SRT was considerably reduced after controlling for auditory working memory. By contrast, even when controlling for auditory working memory and frequency discrimination, beat sensitivity still strongly correlated with SRT in both unmodulated and modulated noise and moderately correlated with masking release.

**TABLE 4.**
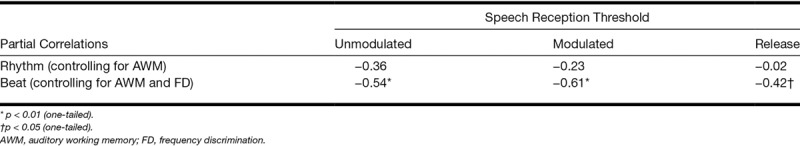
Partial correlation coefficients for the relationships between predictor variables and the three speech-in-noise measures, controlling for AWM and FD

## DISCUSSION

In this study, we used a correlational design to understand more about the link between musical experience and speech-in-noise perception. Specifically, we focused on sensitivity to beat, melody, and rhythm. Of these, beat sensitivity was a notably strong predictor for performance on the Matrix Sentence Test and remained so even after factoring out potentially relevant influences from auditory working memory and frequency discrimination.

### Role of Music Training in Enhancing Speech-in-Noise Perception

While many studies, with very few exceptions (see Ruggles et al. 2014; Boebinger et al. 2015), have demonstrated a benefit of musical training for speech-in-noise listening, they have involved the comparison of highly trained and untrained groups of listeners. It is, therefore, unclear how much training is needed to observe enhanced speech-in-noise perception. In this study, we indexed music experience using the Goldsmiths’ Musical Sophistication Index (Gold-MSI). This questionnaire was specifically developed to measure “the musicality of nonmusicians” (Müllensiefen et al. 2014) and included items assessing both formal training and informal musical experience. Our participants were purposely chosen to have a broad range of musical experience and, reflecting this strategy, scores on the Gold-MSI ranged from 10 (very little experience) to 58 (a high level of experience). Significant linear correlations were observed between musical experience and SRT for both modulated and unmodulated noise maskers. These findings suggest it is not necessary to have intense lifelong musical training to observe enhanced speech perception when listening in noise. In the context of auditory habilitation, the findings suggest that observable benefits for speech-in-noise perception will be observed with practically feasible “doses” of musical training.

### Beat Perception: A Mechanism for Tuning Attention?

The primary aim of this study was to identify which aspects of musicality (sensitivity to beat, rhythm, or melody) may be relevant to the reported musician enhancement for speech-in-noise perception and amenable to training.

Rhythm and beat, but not melody, both associated positively with SRTs. The effect was notably stronger for beat sensitivity, and it remained significant even after partialling out contributions from auditory working memory and frequency discrimination. Slater and Kraus (2016) have previously shown how rhythm sensitivity associates with better performance on the QuickSIN, an open-set sentence test in 4-talker babble, (Killion et al. 2004), but not with the words-in-noise (WIN) test (Wilson et al. 2007), also using a 4-talker babble. Slater and Kraus offered three possible explanations for their findings. Sensitivity to rhythm may help listeners to (a) detect word boundaries (Smith et al. 1989), (b) establish a background invariant neural representation of the signal through enhanced synchronization of low-frequency oscillators to the slow temporal modulations of speech (e.g. Ding & Simon 2013), or (c) eliminate candidate word sequences to support bootstrapping into higher levels of linguistic processing (e.g. Schmidt-Kassow & Kotz 2009b). The association between rhythm sensitivity and the QuickSIN test, but not the WIN test, potentially argued in favor of this latter explanation.

In the present study, we observed a stronger association with beat (the underlying isochronous pulse) than with rhythm (the percept created through variations in stresses and durations of the different notes in a melody line). The association with beat sensitivity was also apparent for the derived measure—modulation masking release. This latter association suggests an additional potential mechanism, whereby sensitivity to beat may enhance dip-listening leading to better SRTs in listeners with musical experience. The question is, how does sensitivity to beat support dip-listening? We hypothesize that it may happen through automatic entrainment to the underlying beat in the speech signal.

Entrainment to a regular beat is a fundamental musical skill and an innate human ability that has been observed in infants (Honing 2012). Beat perception requires that listeners encode the timing of a beat (regular pulse) and form predictions about when the next beat will occur. We inherently vary in how, and how well, we perceive a beat (Grahn & McAuley 2009; Thompson et al. 2015), but beat perception is amenable to training (Slater et al. 2013). In the BAT, automatic entrainment to the beat enabled listeners to compare the superimposed, experimental beep timings with a predicted beat in the music. The Matrix Sentence Test involved the detection and perception of five words per sentence, where the first syllable of each word was presented on-beat according to a simple isochronous beat. Automatic entrainment to this beat may have established a form of anticipatory attention (Jones et al. 2002) enabling listeners to predict the onset of each syllable and thus exploit cues available through dip-listening to achieve better SRTs. While our choice of speech-in-noise test may have resulted in an enhanced correlation with beat perception, the strength of the relationship suggests it is unlikely to be the whole story.

Speech, unlike music, does not necessarily contain an isochronous beat (Schön & Tillmann 2015). Nonetheless, it has a metric structure which it derives from the combinations of strong and weak syllables making up individual words within sentences. Listeners are able to tap along to this regularity, much as they would to a beat in music (Lidji et al. 2011). Furthermore, the metric structure of speech has been shown to facilitate predictions about when the next strong syllable will occur in streams of words or sentences (Pitt & Samuel 1990; Quené & Port 2005). Such predictions may focus attention to points in time when important parts of the signal are to be expected (Cason & Schön 2012; Schön & Tillmann 2015) and may facilitate speech perception when listening in noise. Trained musicians with an enhanced sensitivity to the underlying metric structure of speech would, therefore, be expected to have better SRTs. However, the validity of this hypothesis would need to be further explored by perturbing temporal expectations about the underlying beat to assess the impact on SRT.

### Measurement of Beat Versus Rhythm Sensitivity and Its Impact on Study Conclusions

Beat and rhythm are closely related and together describe the temporal organization of music, where beat is pre-eminent (Peretz & Zatorre 2005). One would expect measures of these two skills to correlate highly. Yet we only observed a modest correlation. Furthermore, a strong correlation was observed between measures of rhythm and melody sensitivity, but not beat and melody sensitivity. The pattern of correlations observed reflects key differences in the design of the tests used to gauge beat and rhythm sensitivity.

The beat test involved a direct comparison between strings of beeps superimposed on a melodic phrase. In addition to ability to automatically entrain to the beat, performance on this test requires a capacity to segregate competing streams of auditory input and attend separately to each. A similar capacity is required when listening to speech-in-noise. The strong correlations between beat processing and speech-in-noise listening may reflect not only effects due to anticipatory attention but also effects due to differences in ability to segregate competing streams of inputs (Anderson & Kraus 2011).

The rhythm and melody subtests of the Musical Ear Test involved holding one phrase in memory while listening and comparing it with a second phrase. As such, in addition to musicality, test performance would also reflect individual differences in auditory working memory capacity. Underlining this point, it is notable how musical experience does not correlate as strongly as auditory working memory with performance on these tests. This contrasts with performance on the BAT, where musical experience is a stronger predictor of performance.

### Auditory Working Memory and Frequency Discrimination in Supporting Speech-in-Noise Perception

It has been suggested that musical training–related enhancements in both auditory working memory and frequency discrimination contribute to the better speech perception skills associated with highly trained musicians (Kraus et al. 2012; Parbery-Clark et al. 2009b). Our study did not offer compelling evidence to support this. While we saw clear correlations with the musical skills tested, none of the correlations between auditory working memory and any SRT reached significance. In the case of the spectral measures (melody perception and frequency discrimination), a moderate correlation was only observed between SRT in amplitude-modulated noise and frequency discrimination. Our findings largely replicate those of Ruggles et al. (2014) and more recently Slater and Kraus (2016). Ruggles et al. failed to demonstrate an association with SRT and frequency discrimination. Slater and Kraus (2016) failed to find an association between SRT measured using the QuickSIN and either auditory working memory or melody perception as measured using the Musical Ear Test.

Working memory is often implicated in supporting speech-in-noise perception with, or without, musical training (e.g., Füllgrabe et al. 2015; Heinrich & Knight 2016; Wayne et al. 2016). We may not have observed a correlation between auditory working memory and SRT, because our speech-in-noise test was not sufficiently demanding in terms of working memory resources. In support of this, it is notable how, if an effect for memory is observed, it is more likely to be with tests involving sentence repetition, like the HINT or QuickSIN, than with tests limited to single-word perception, like the WIN (Parbery-Clark et al. 2011b). Alternatively, we may have added measurement noise through combining the forward and backward digit span measures into a single measure of auditory working memory, since these are thought to index serial memory and executive function, respectively, which are viewed as separate components of working memory (Rosenthal et al. 2006).

With respect to frequency discrimination, it has been suggested that musicians’ better speech-in-noise perception is due to enhanced neural encoding of periodicity (cyclical repetitions at a particular frequency) as gauged by their excellent frequency discrimination abilities (Parbery-Clark et al. 2009b). We saw little evidence for this association. The frequency discrimination task itself is highly susceptible to auditory experience (Amitay et al. 2005), so relatively little musical training seems to have a positive impact on frequency discrimination thresholds (Barry et al. 2013). Likewise, relatively little training on frequency discrimination will result in thresholds equivalent to those of expert musicians (Micheyl et al. 2006). Musical training may enhance performance on frequency discrimination tasks by providing the listener with some form of perceptual anchor (Ahissar et al. 2006) or stimulus label (Roebuck et al. 2016) to support decisions about perceptual differences between relatively similar auditory inputs.

## CONCLUSIONS

There is great interest in using music training to remediate difficulties with listening in noise. In contrast with many studies, we deliberately included participants with a wide range of musical experience, yet still replicated previous findings of an association between musical training and better SRTs. This suggests that relatively modest amounts of training may have a positive impact on speech perception.

Although causation cannot be inferred from a correlation analysis, the pattern of associations observed here could provide insight into the link between musical training and speech-in-noise perception. Specifically, the findings suggest that the enhancements in SRT reflect entrainment to the underlying beat in the speech signal leading to more efficient dip-listening through anticipatory attention.

As a minor part of the study, we also assessed the feasibility of using the Matrix Sentence Test with amplitude-modulated maskers to assess influences of musical training on SRT. We found that a masker with an 80% modulation depth was more sensitive to individual differences in SRT than one with a 60% modulation depth. This kind of closed-set speech-in-noise test combined with the right masker may be suitable for use in training studies, since it offers many benefits, including reliability of SRT measurement across multiple administrations.

## ACKNOWLEDGMENTS

The research was funded with Medical Research Council intramural funding grant U135097130. J.G.B. was funded through the Nottingham University Hospitals National Health Service Trust Flexibility and Sustainability Fund.

## Supplementary Material

## Footnotes

1 Following Füllgrabe et al. (2015), the term “unmodulated” is used instead of “steady state” since “notionally” steady noise produces masking primarily through amplitude fluctuations thereby reducing both the relative energy and intelligibility of the signal (Stone et al. 2012).
