# Supplementary material for: Sensitivity to Melody, Rhythm, and Beat in Supporting Speech-in-Noise Perception in Young Adults
Source: Ear Hear. 2019 Feb 27;40(2):358–67. doi: 10.1097/AUD.0000000000000621 (PMC6400450; doi:10.1097/AUD.0000000000000621)
Supplement: Supplementary file 1 [file aud-40-358-s001.docx]

# Supplemental Appendix


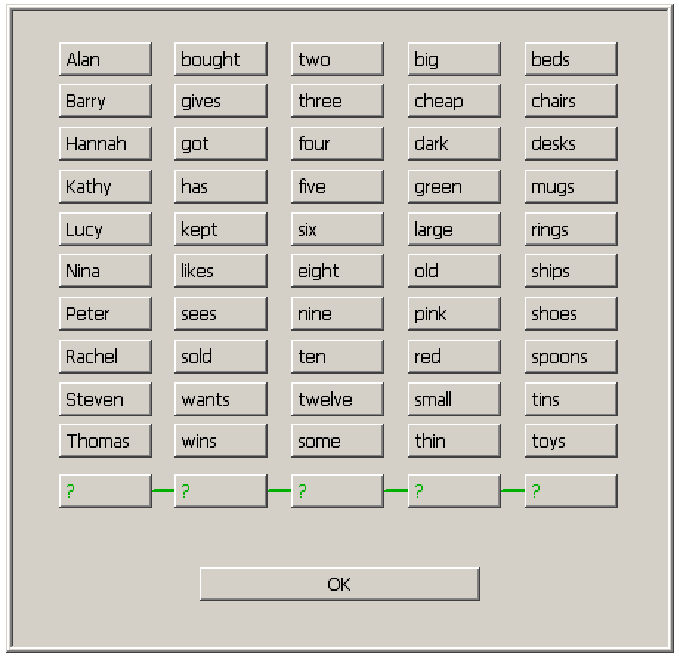


Screenshot of the response screen showing the matrix of possible words that make up the sentences in the Matrix Sentence Test. Each sentence consists of one word from each column, for example: ‘Nina kept three small desks’.
